# Effect of minimally invasive treatment for intrabony defects using Bio-Oss® collagen

**DOI:** 10.6026/973206300200877

**Published:** 2024-08-31

**Authors:** Kashish Luthra, Vandana Sarangal, Sahib Tej Singh, Simranjit Kaur, Vasudha Suchdev

**Affiliations:** 1 Department of Periodontics, Sri Guru Ram Das Institute of Dental Sciences and Research, Amritsar, India

**Keywords:** Bio-Oss®, collagen, minimally invasive surgical technique, periodontitis, regenerative

## Abstract

The potential of Bio-Oss® collagen xenograft for treating intrabony defects using minimally invasive surgical methods is of interest. Hence, 30 defect sites in individuals with mild to severe chronic periodontitis were investigated. Participants were randomly divided into two groups of 15 each. Group A underwent regenerative MIST (minimally invasive surgical technique) with a bovine-derived xenograft, while Group B received only MIST. Periodontal parameters, including plaque index, gingival index, probing pocket depth, clinical attachment levels, and intrabony defect fill, were evaluated at three and six-month intervals. Significant improvements in periodontal parameters were observed in both groups, with Group A showing significantly better results.

## Background:

Periodontitis is defined by gingival inflammation, the creation of periodontal pockets, and the loss of connective tissue attachment and alveolar bone surrounding the impacted teeth [1]. Periodontitis has a complex etiology. Subgingival dental biofilm, which includes the alveolar bone and periodontal ligament, induces an inflammatory and immunological response in a susceptible host, which ultimately leads to the permanent destruction of the periodontium [2]. Over many years, the illness typically exhibits few or minor symptoms, which the patient frequently fails to, recognize or appropriately classify. Insufficient knowledge may result in seeking dental care when the condition has progressed and more extensive therapeutic treatments are required, with a worsening prognosis for tooth retention [3]. Periodontitis has been connected to a range of systemic health problems, such as adverse pregnancy outcomes, heart disease, type 2 diabetes mellitus, respiratory diseases, life-threatening pneumonia in hemodialysis patients, chronic kidney disease, and metabolic syndrome. [4]. Gaining access to disease regions, lowering pocket depth, stopping further disease development, and finally repairing the lost periodontal tissues owing to the disease process remains the mainstay of periodontal therapy [5]. In the trial, minimally invasive surgery is combined with bovine derived xenograft, Bio-Oss® collagen to fill defects and improve periodontal metrics [6]. Therefore, it is of interest to report the developments of new attachments to achieve periodontal regeneration.

## Material and Methods:

Patients with moderate to severe chronic periodontitis who visited the Department of Periodontology at our research institution had 30 defect sites investigated in this randomized clinical investigation. Initially, the ethics committee of the institution reviewed and approved the research protocol. Following ethical clearance, the study was disclosed to all patients, and each participant's written agreement was sought before they could be included in the study. The research was carried out in compliance with the guidelines provided in the 1975 Helsinki Declaration, which was updated in 2013 [7].

## Criteria for selection of patients:

## Inclusion criteria:

Patients suffering from moderate to severe chronic periodontitis typically present with intrabony flaws characterized by probing pocket depths (PD) and clinical attachment levels (CAL) of ≥5mm. Additionally, radiographic evidence reveals intra-bony defects of ≥3mm. Suitable candidates for treatment are those who demonstrate cooperative behavior and maintain acceptable oral hygiene practices. These patients must also be willing to sign the consent form to proceed with the necessary periodontal interventions.

## Exclusion criteria:

Excluded from the study are patients with any systemic disease impacting periodontal health, those who have had periodontal therapy within the last six months, alcoholics, smokers, tobacco chewers, drug addicts, expectant mothers, and nursing moms.

## Study design:

Individuals who satisfied the requirements for inclusion and exclusion were divided into two groups: Group A and Group B. In Group A, 15 defect sites were treated using a minimally invasive surgical technique (MIST) with xenografts (Bio-Oss® collagen) for filling the defects. In Group B, 15 defect sites were treated with MIST alone. Clinical data were collected at 3 and 6 months post-surgery, and X-ray examinations were conducted at these intervals. The clinical parameters assessed included gingival index, pocket probing depth, and clinical attachment level. Radiographic evaluations of the intrabony defects were also analyzed at baseline, 3 months, and 6 months. Participants were instructed to rinse with 0.12% chlorhexidine digluconate before the procedure. After achieving adequate anesthesia with 2% lignocaine and epinephrine, crevicular incisions were made on the facial side to the tip of the interdental papilla using a Bard-Parker handle and No. 15 blade. For MIST, intra-sulcular incisions were made, and a muco-periosteal flap was reflected on the buccal side of the defect with a periosteal elevator. After flap reflection and exposure of the bony defect, Gracey curettes and 4R-4L Columbia universal curettes (Hu-Friedy) were used to perform thorough degranulation of contaminated tissue. The site was then irrigated with normal saline. In Group A, Bio-Oss® collagen was gradually introduced into the bony defect using a bone graft carrier and carefully packed. In Group B, the defect site was exclusively treated with MIST. The surgical flaps were repositioned to their original levels and secured with an interdental direct suture using 3-0 non-absorbable braided silk to ensure primary closure. A non-eyelet periodontal dressing (Coe-pak®) was applied over the site. Data were gathered, processed, and analyzed using SPSS version 17.0. The normality of the data was assessed using the Kolmogorov-Smirnov and Shapiro-Wilk tests. Gender distribution was compared with chi-square tests. Periodontal parameters were analyzed for mean values, standard deviations, and percentage changes using t-tests: paired t-tests for within-group comparisons and unpaired t-tests for between-group comparisons at baseline, 3 months, and 6 months. Patients were required to attend follow-up appointments and were consistently reminded of their oral hygiene practices. This emphasis on patient adherence to treatment highlighted the need for further research to determine the effectiveness of such reminders in improving patient outcomes.

## Result:

The study involved 30 participants with moderate to severe chronic periodontitis, with a mean age of 50.5 years (standard deviation 1.36). The cohort comprised 55% females and 45% males. At baseline, Group A, which received MIST with Bio-Oss® collagen xenograft, had a mean plaque index score of 0.80 ± 0.19. This score decreased to 0.67 ± 0.15 at 3 months and further to 0.59 ± 0.15 at 6 months. Conversely, Group B, which underwent MIST alone, had a baseline plaque index score of 0.84 ± 0.22. This score reduced to 0.75 ± 0.21 at 3 months and to 0.67 ± 0.19 at 6 months (Table 1). When mean plaque index scores were compared between groups A and B. The differences were determined to be statistically non-significant (p>0.05) at baseline, 3 and 6 months after surgery. Both groups demonstrated changes in plaque index three and six months post-operatively. The mean value of gingival index score in group A was 1.15 ± 0.23 which reduced to 0.96 ± 0.18 at end of 3 months which further reduced to 0.75 ± 0.21 at the end of 6 months. Whereas, the mean value of gingival index score in group B was 1.20 ± 0.27 which reduced to 0.99 ± 0.26 at end of 3 months which further reduced to 0.81± 0.31 at the end of 6 months (Table 2). On comparison of mean values of gingival index scores between group A and B, the differences were found to be non-significant (p>0.05), at baseline, 3 and 6 months postoperatively. At 3 months post-treatment, Group A exhibited a mean reduction in probing pocket depth (PPD) of 3.35 ± 0.68 mm. By 6 months, the mean reduction increased to 3.62 ± 0.78 mm, with a change of 0.27 ± 0.31 mm observed between 3 and 6 months. The reduction from baseline to 3 months and from baseline to 6 months was statistically highly significant, while the change between 3 and 6 months was also significant. In Group B, the mean PPD reduction at 3 months was 2.00 ± 0.76 mm, which increased to 2.12 ± 0.76 mm by 6 months. The reduction at 6 months was statistically significant, but the change between 3 and 6 months (0.12 ± 0.18 mm) was not significant (Table 3). Therefore, indicating maximum change in PPD from baseline to 3 months and the further reduction continues till 6 months in both the groups. Change from 3 to 6 months was more in group A then group B. It was observed that at 3 months' post-treatment, the values showed a mean gain in the clinical attachment level by 3.74 ± 0.77 mm and 1.97 ± 1.02 in group A and B, respectively.

Further gain was observed by 4.18 ± 0.85 and 2.10 ± 0.93 at end of 6 months in both groups (A and B) respectively indicating a statistically highly significant difference (p<0.001) from baseline. From the mean values it is evident that significant clinical attachment gain was observed in both group A and group B. However, the CAL gain between 3 to 6 months in group A and group B was 0.43 ± 0.32mm and 0.13±0.12 mm respectively depicting significant change. Maximum CAL gain was observed between baseline and 3 months' indicative of attachment level gain which also continued between periods of 3-6 months post-operatively (Table 4).

When comparing the two groups, changes in clinical attachment level (CAL) were found to be highly significant from baseline to both 3 and 6 months. Group A demonstrated a greater CAL gain compared to Group B. Radiographic assessments revealed significant changes from baseline to 3 and 6 months. In Group A, the changes were 1.44 ± 0.52 mm at 3 months and 2.68 ± 0.61 mm at 6 months. In Group B, the changes were 1.20 ± 0.67 mm at 3 months and 1.33 ± 0.71 mm at 6 months. These differences were statistically highly significant (p < 0.001) from baseline. However, the change between 3 and 6 months was 1.23 ± 0.65 mm in Group A and 0.13 ± 0.21 mm in Group B, indicating a significant change in Group A and a non-significant change in Group B. The change from baseline to 3 months was greater than the change from 3 to 6 months, indicating that the maximum bone fill occurred earlier. Bone regeneration continued up to 6 months (Table 5). On comparison between the groups the change was found to be highly significant other than at baseline which was found to be non-significant. The percentage of bone fill was found to be 28.62 % and 22.36 % in the first 3 months which was highly significant from baseline in group A and group B respectively. However, the bone fill percentage when compared between both the groups showed non- significant change percentage. However, at the end of 6 months the bone formation was more in group A with percentage of 53% and less in group B with percentage of 24.84% indicating highly significant difference. However, bone formation from 3 to 6 months was more in group A (34.14%) than in group B (3.18%) which in comparison between both the groups was found to be highly significant. Bone formation was high from baseline till 3 months and continued till 6 months in both the groups indicative of its regenerative potential.

## Discussion:

Plaque is thought to be the primary cause of initiation of periodontal disease process and severity of periodontal infection. The results of full mouth disinfection, which is the first line of treatment for periodontal infections and is regarded as the gold standard for treating periodontal disease, may be the cause of the observed decrease in plaque index scores in groups A and B in the current study. The bacterial biofilm, which plays a critical role in the initiation and recurrence of disease, is disturbed and inflammation is reduced by mechanical debridement. Consequently, it is essential to regularly and continuously disrupt these biofilms in order to prevent and treat oral illnesses. A further advantage of SRP is that it changes the surrounding environment where the pathogenic species reside [8, 9]. According to McCarney, the Hawthorne phenomenon or the mandated dental hygiene training were attributed for the decrease in plaque score. The Hawthorne effect is a type of reactivity in which participants adjust or enhance a behavior that is being assessed experimentally when they become aware that they are being evaluated [10]. In the Greenstein G Study, throughout the course of the therapy, there was a decrease in the gingival index in both group A and B. This decrease in plaque scores could be linked to a decrease in gingival inflammation, which would then cause tissue shrinking [11]. Lindhe *et al.* identified the critical probing depths for scaling and root planing (2.9 ± 0.4 mm) and modified Widman flap surgery (4.2 ± 0.2 mm) 9 . Their findings suggest that a non-surgical approach is preferable for patients with shallow probing depths, while surgical treatment may result in more attachment gain in patients with deep pockets > 4.2 mm 8,9 [12]. At three and six months from baseline, both group A and group B showed a reduction in probing pocket depth. One possible explanation for the decreased pocket depth is that the connective tissue re-organizes when the inflammatory condition resolves. According to Lekovic *et al.* the primary reason for the greater reduction in probing pocket depth in Group A compared to Group B following therapy is the decrease in inflammation and the shrinking of the pocket wall. Additionally, it is proposed that the implantation of graft material into the defect site may alter the consistency of the gingival tissue, thereby limiting the penetration of the periodontal probe without necessarily resulting in any gain in clinical attachment [13]. According to Camargo PM *et al.*, there is reduction in probing pocket depth due to presence of graft material in intrabony defect sites which not only lead to bone formation due to its osteo-conductive properties but also leads to improvement in tissue tone which further makes it difficult for periodontal probe penetration. There is new attachment due to the presence of regenerative materials which leads to formation of long junctional epithelium between root surface and gingival epithelium. Additionally, Bio-Oss® collagen serves as a scaffold to assist in the development of new tissue, which is then replaced by the host tissues. Addition of 10% porcine collagen makes it easy to handle and formable. Also it is quick to apply due to the addition of collagen. The material has low resorption rate and good adherence to the tissues; hence tissue remains stable in volume [14]. According to Tonetti *et al.*, clinical measurement of attachment level, first presented by Ramfjord, has evolved into a gold standard for assessing clinical response in periodontitis treatment. Clinical attachment level has become a significant outcome variable in regenerative investigations due to the nearly perfect positive connection seen between the gain in clinical attachment level and the increase in bone height [15]. Both group A and group B patients showed improvements in their clinical attachment levels at intervals of 3 and 6 months. True periodontal regeneration through new attachment may have contributed to this gain in clinical attachment. More gain was observed in group A when compared to group B. The possible explanation could be because of the physical characteristics of Bio-Oss®, which help to stabilize blood clots and isolate proper connective tissue and gingival epithelial cells from the root surface and defect area. With 10% porcine collagen added, Geistlich Bio-Oss Collagen® is 90% Bio-Oss® granules. Bio-Oss® is the most advanced biomaterial in regenerative dentistry, supported by scientific evidence. This graft becomes more pliable and manageable when combined with ultra-purified porcine collagen, improving its handling qualities [16]. Since alveolar bone is a crucial part of the periodontium, defect fill is a desired result of periodontal regeneration therapies. Even while there may be new connective tissue formation, clinical healing of osseous lesions does not qualify as regeneration if there is no new bone. Thakkalapati *et al.* concluded that radio-graphically first substantiation of bone formation was seen as early as 3 months with almost complete bone fill by 6 months [17]. Bone fill was observed more in group A than group B because of the fact that Bio-Oss® collagen is made up of 90% Bio-Oss® granules and 10% collagen which is manufactured by extracting proteins from bovine bone, which produces a trabecular hydroxyapatite structure resembling human cancellous bone and has the capacity to promote bone formation due to its osteo-conductivity, addition of 10 % porcine collagen to the graft stimulates rapid vascularization and epithelial regeneration, which supports bone regrowth could be one reason for the noteworthy defect fill. In the current study, all clinical and radiographic measures recorded in Groups A (MIST +Bio-Oss®) and B (MIST) showed favorable responses. Individually, both groups displayed statistically significant defect fill. They also demonstrated gain in clinical attachment level and a statistically significant decrease in probing pocket depth. Despite additional benefits from the xenograft, MIST performed either in combination or exclusively showed promising results with less tissue morbidity, better patient compliance with less time required for surgery However, further studies with larger sample size and longer follow-up period are suggested to more accurately identify and measure the morphology of bone defects and to determine the long term effects of Bio-Oss® collagen bone graft and minimally invasive surgical technique in the treatment of intrabony defects.

## Conclusion:

The use of Bio-Oss® Collagen represents a novel approach towards periodontal regeneration and can be used routinely in clinical practice. Thus, minimally invasive surgical technique exhibited significant improvement both with and without bovine derived xenograft Bio-Oss® collagen.

## Figures and Tables

**Table 1:** Comparison of Mean Change In Plaque Index Scores In Group A (MIST + Bio-Oss® Collagen) and Group B (MIST)

**GROUP**	**Group A (N=15)**		**Group B (N=15)**		**Group A vs Group B**
	**Mean ± SD**	**P-value**	**Mean ±SD**	**P-value**	**P value**
Baseline-3 months	0.12 ± 0.08	<0.001	0.09± 0.07	<0.001	0.265
Baseline-6 months	0.20 ± 0.10	<0.001	0.17 ± 0.07	<0.001	0.295
3-6 months	0.08±0.07	0.001	0.08±0.06	0.001	0.895

**Table 2:** Comparison of Mean Change In Gingival Index Scores In Group A (MIST +Bio-Oss® Collagen) and Group B (MIST)

**Group**	**Group A (N=15)**		**Group B (N=15)**		**Group A Vs Group B**
	**Mean ± SD**	**P-value**	**Mean ±SD**	**P-value**	**P value**
Baseline-3 months	0.19± 0.10	<0.001	0.21± 0.13	<0.001	0.599
Baseline- 6 months	0.40 ± 0.09	<0.001	0.39 ± 0.21	<0.001	0.956
3-6 months	0.21± 0.09	<0.001	0.18±0.14	<0.001	0.553

**Table 3:** Comparison of Mean Change in Probing Pocket Depth (PPD in mm) In Group A (MIST +Bio-Oss® Collagen) and Group B (MIST)

**Group**	**Group A (N=15)**		**Group B (N=15)**		**Group A vs Group B**
	**Mean ± SD**	**P-value**	**Mean ±SD**	**P-value**	**P-value**
Baseline-3 months	3.35±0.68	<0.001	2.00± 0.76	<0.001	<0.001
Baseline - 6 months	3.62 ± 0.78	<0.001	2.12 ± 0.76	<0.001	<0.001
3-6 months	0.27±31	0.005	0.12±0.18	0.22	0.123

**Table 4:** Comparison of Mean Change in Clinical Attachment Level (CAL) In Group A (Mist +Bio-Oss® Collagen) and Group B (Mist)

**Group**	**Group A (N=15)**		**Group B (N=15)**		**Group A vs Group B**
	**Mean ± SD**	**P-value**	**Mean ±SD**	**P-value**	**P-value**
Baseline-3 months	3.74.± 0.77	<0.001	1.97 ± 1.02	<0.001	<0.001
Baseline-6 months	4.18 ± 0.85	<0.001	2.10 ± 0.93	<0.001	<0.001
3-6 months	0.43±0.32	<0.001	0.13±0.12	0.001	0.002

**Table 5:** Comparison of Mean Change In Intrabony Defect Fill (In mm)

**Group**	**Group A (N=15)**		**Group B (N=15)**		**Group A vs Group B**
	**Mean ± SD**	**P-value**	**Mean ±SD**	**P-value**	**P-value**
Baseline-3 months	1.44 ± 0.52	<0.001	1.20 ± 0.67	<0.001	0.277
Baseline-6 months	2.68 ± 0.61	<0.001	1.33 ± 0.71	<0.001	<0.001
3-6 months	1.23±0.65	<0.001	0.13±0.21	0.29	<0.001
